# Describing skin health and disease in urban-living Aboriginal children: co-design, development and feasibility testing of the Koolungar Moorditj Healthy Skin pilot project

**DOI:** 10.1186/s40814-023-01428-6

**Published:** 2024-01-11

**Authors:** Bernadette M. Ricciardo, Heather-Lynn Kessaris, Noel Nannup, Dale Tilbrook, Brad Farrant, Carol Michie, Lorraine Hansen, Richelle Douglas, Jacinta Walton, Ainslie Poore, Alexandra Whelan, Timothy C. Barnett, Prasad S. Kumarasinghe, Jonathan R. Carapetis, Asha C. Bowen

**Affiliations:** 1 University of Western Australia, Crawley, WA Australia; 2 Wesfarmers Centre of Vaccines and Infectious Diseases, Telethon Kids Institute, Nedlands, WA Australia; 3 Fiona Stanley Hospital, Murdoch, WA Australia; 4 Perth Children’s Hospital, Nedlands, WA Australia; 5 Telethon Kids Institute, Nedlands, WA Australia; 6 Maalingup Aboriginal Gallery, Caversham, WA Australia; 7 Derbarl Yerrigan Health Services Aboriginal Corporation, East Perth, WA Australia; 8 Marshall Centre for Infectious Diseases Research and Training, School of Biomedical Sciences, University of Western Australia, Nedlands, WA Australia

**Keywords:** Co-design, Pilot, Skin health, Skin disease, Dermatology, Children, Adolescents, Aboriginal, Urban

## Abstract

**Background:**

Indigenous children in colonised nations experience high rates of health disparities linked to historical trauma resulting from displacement and dispossession, as well as ongoing systemic racism. Skin infections and their complications are one such health inequity, with the highest global burden described in remote-living Australian Aboriginal and/or Torres Strait Islander (hereafter respectfully referred to as Aboriginal) children. Yet despite increasing urbanisation, little is known about the skin infection burden for urban-living Aboriginal children. More knowledge is needed to inform service provision, treatment guidelines and community-wide healthy skin strategies. In this pilot study, we aimed to test the feasibility and design of larger multi-site observational studies, provide initial descriptions of skin disease frequency and generate preliminary hypotheses of association.

**Methods:**

This project has been co-designed with local (Noongar) Elders to provide an Australian-first description of skin health and disease in urban-living Aboriginal children. In collaboration with an urban Aboriginal Community Controlled Health Organisation (Derbarl Yerrigan Health Service), we conducted a week-long cross-sectional observational cohort study of Aboriginal children (0–18 years) recruited from the waiting room. Participants completed a questionnaire, skin examination, clinical photos, and swabs and received appropriate treatment. We assessed the feasibility and impact of the pilot study.

**Results:**

From 4 to 8 October 2021, we recruited 84 Aboriginal children of whom 80 (95%) were urban-living. With a trusted Aboriginal Health Practitioner leading recruitment, most parents (or caregivers) who were approached consented to participate. Among urban-living children, over half (45/80, 56%) of parents described a current concern with their child’s skin, hair and/or nails; and one-third (26/80, 33%) reported current itchy skin. Using a research-service model, 27% (21/79) of examined urban-living participants received opportunistic same-day treatment and 18% (14/79) were referred for later review.

**Conclusions:**

This co-designed pilot study to understand skin health in urban-living Aboriginal children was feasible and acceptable, with high study participation and subsequent engagement in clinical care observed. Co-design and the strong involvement of Aboriginal people to lead and deliver the project was crucial. The successful pilot has informed larger, multi-site observational studies to more accurately answer questions of disease burden and inform the development of healthy skin messages for urban-living Aboriginal children.

**Supplementary Information:**

The online version contains supplementary material available at 10.1186/s40814-023-01428-6.

## Key messages regarding feasibility

- 1. What uncertainties existed regarding the feasibility?

Several uncertainties existed regarding the feasibility of this study, e.g. “How do we best promote the project?” “Can we recruit adequate numbers of eligible participants from within the setting of an urban Aboriginal Community Controlled Health Organisation (ACCHO)?” “Are the study procedures acceptable to and suitable for participants and their families?” “Are the data collection tools optimised for usability by the screening team and statistical analysis?” Does the research team have the resources and capacity to conduct the study?” “Is the research-service model desirable for participants and their families?”

- 2. What are the key feasibility findings?

This co-designed pilot study proved feasible and acceptable, with high participation in the study and subsequent engagement in clinical care observed. There was a steady increase in participant numbers over the course of the week, consistent with cultural protocol and awareness. This was highlighted to the non-Aboriginal researchers by the Aboriginal researchers as a likely occurrence and played out as expected during the pilot study. The involvement of a trusted Aboriginal Health Practitioner was key to recruiting Aboriginal families, achieving a sample size of 84 participants and surpassing the estimated pilot size of 50, that was originally thought to be feasible in a 1-week study. The ACCHO setting ensured cultural safety and facilitated the research-service model, whereby over a quarter of participants received opportunistic same-day management of their skin condition. Co-design and the strong involvement of Aboriginal people to lead and deliver the project was crucial.

- 3. What are the implications of the feasibility findings to the design of the main study?

Following feedback from the Elder and ACCHO co-researchers, minor adjustments were made to the protocol to reflect ongoing Elder co-researcher cultural guidance and community consultation; expand the scope of promotion; adjust the data collection tools to better inform generalisability, enhance validity and further explore the significant disease associations observed in the pilot; and optimise research document usability and statistical analysis for the screening team. The pilot has informed a successful funding application to conduct the needed larger, multi-site observational studies where the updated protocol will be used to more accurately answer questions of disease burden and inform the development of healthy skin messages for urban-living Aboriginal children. Walking together in the co-design model invites a learning mindset from all participating researchers. This learning together was a significant part of the pilot study and has contributed overall to the feasibility and cultural acceptability of ongoing work.

## Background

Skin health is an important aspect of overall health including appearance, well-being and confidence, due to the skin being the largest and only visible organ of the body. Different populations have different experiences of skin health and disease, and this informs targeted healthy literacy and health promotion resources. Having identified knowledge gaps in skin health for urban-living Australian Aboriginal and/or Torres Strait Islander (hereafter respectfully referred to as Aboriginal) children, we piloted a cross-sectional, observational cohort study to inform future research and health promotion activities.

### Infectious skin diseases

Indigenous children face higher rates of health disparities than their non-Indigenous peers globally, linked to historical trauma resulting from the devastating effects of colonisation, dispossession and loss of connection to traditional lands, language, family and culture [1].

Skin infections are one example of this, where the highest burden in the world is well documented in remote-living Aboriginal children [2]. The most significant of these are bacterial skin infections (BSI) with *Staphylococcus aureus* (*S. aureus*) and *Streptococcus pyogenes* (*S. pyogenes*) causing impetigo, cellulitis and abscesses; and scabies [2, 3] At any time, almost half of all Aboriginal children in remote Australia will have impetigo and up to one-third will have scabies [4, 5]. These often painful and itchy skin conditions adversely affect wellbeing and self-image, and can lead to serious complications including bone and joint infections, sepsis, post-streptococcal glomerulonephritis and rheumatic heart disease. These infections have a high burden on individuals, communities and the health system, both in primary and tertiary care [6].

Whilst much is known about remote-living Aboriginal children, a knowledge gap exists regarding the skin infection burden for urban-living Aboriginal children. This is despite the rate of urbanisation for Indigenous people increasing globally. In our context, more than 60% of approximately 40,000 Aboriginal children (0–17 years) currently reside in urban settings (major cities and regional areas) [7, 8].

Linked hospitalisation data found hospitalisation rates for skin infections (abscess, cellulitis, impetigo and scabies) were 10 times higher for urban-living Aboriginal children than their non-Aboriginal peers [6]. This likely underestimates the true burden of skin infection in urban-living Aboriginal children, as it only captures the severe end of the disease burden found in hospitals, and not the primary care data where most skin infections present. A New Zealand study investigating Indigenous children reported that an estimated 14 primary care cases occurred for every skin infection resulting in hospitalisation [9]. A further limitation to coded-hospitalisation datasets is “normalisation” or the documented under-diagnosis associated with the high ongoing burden of skin infections [10].

Another source of data on urban-living children is carer surveys. An older study self-reported recurring skin infections in 720/10,200 (7.1%) of urban-living Aboriginal children; with limitations being normalisation, knowledge and the ability to identify and recall skin infections by the carer [11]. Beyond this, data on the complications of skin infections in urban-living Aboriginal children are poorly understood. One national dataset of paediatric intensive care unit admissions found invasive infections were higher in Aboriginal compared to non-Aboriginal children, particularly *S. aureus* sepsis secondary to untreated skin infections [12, 13].

### Non-infectious skin diseases

Eczema (or atopic dermatitis) is another common skin condition where there is a paucity of data on the burden for urban-living Aboriginal children. The population prevalence of eczema in Australia is estimated to be 20% in 1-year-olds [14] and 16% in 4-year-olds [15]. In two small urban paediatric studies, a history of ‘eczema ever’ was reported in 13–25% of Aboriginal children by 6 years [16, 17]. Eczema and BSI are intertwined, in that poorly managed eczema predisposes to recurrent skin infections; while secondary infection of eczema contributes to more severe disease [18, 19].

Little is known about the burden of other non-infectious skin diseases in Aboriginal Australians, despite skin problems representing 15–18% of primary care consultations [20]. In addition, little is known about dermatologist-diagnosed skin disorders in the urban-living Aboriginal population, with studies revealing rates of attendance to hospital dermatology outpatient appointments for Aboriginal Australians to be lower than population parity [21, 22].

### Aims and objectives

The primary aim of the Koolungar Moorditj Healthy Skin pilot project is to inform the feasibility and design of larger multi-site observational studies. The secondary aims are to provide initial descriptions of skin health and disease frequency in urban-living Aboriginal children recruited from the waiting room of an urban Aboriginal Community Controlled Health Organisation (ACCHO) and to generate preliminary hypotheses regarding skin disease associations. The results of the larger, multi-site observational studies will be the subject of a sequential paper and used to inform service provision, treatment guidelines and to identify sustainable and acceptable community-wide strategies for prevention and treatment.

This study is operational in Perth, Western Australia, Australia where the traditional custodians are the Noongar people. In Noongar language, *koolungar* means children and *moorditj* means good, solid and strong. Our research team has a shared vision to achieve *moorditj healthy skin* for all urban-living Aboriginal children.

## Methods

This manuscript has been reported in accordance with the Consolidated Standards of Reporting Trials (CONSORT) extension to randomised pilot and feasibility trials and the Strengthening the Reporting of Observational Studies in Epidemiology (STROBE) guideline, ignoring items that are not applicable (Additional files 1 and 2) [23–25]. The manuscript also follows the CONSoliDated critERia for strengthening the reporting of health research involving Indigenous Peoples (CONSIDER) statement (Additional file 3) [26].

### Study design

Through a co-design process with Noongar Elders we piloted a cross-sectional, observational, cohort study of urban-living Aboriginal children and adolescents (hereafter ‘children’) recruited from the Derbarl Yerrigan Health Service Aboriginal Corporation (DYHSAC) waiting room [27].

### Co-design process

The Koolungar Moorditj Healthy Skin project was co-designed from the outset, working in collaboration with Noongar Elders at the Telethon Kids Institute (TKI) to determine the interest, scope and importance of skin health for urban-living Aboriginal children. Over several meetings, it was clear that the intersection between healthy skin and a healthy environment was the common ground. From here, two Elders (NN, DT) declared their ongoing support for this work and have continued as co-researchers on the project. It is through this engagement with Noongar Elders that this project has progressed, using the principles of reciprocity, capacity building, respect and community involvement to shape all elements of the protocol; in line with the National Health and Medical Research Council (NHMRC) Indigenous Health Research Criteria (Additional file 4) and TKI Guidelines for the Standards for the Conduct of Aboriginal Health Research [28].

### Study setting

With a priority being a research-service model, we collaborated with DYHSAC to establish a paediatric dermatology outreach clinic. DYHSAC is the oldest and largest ACCHO in WA, with four clinical sites in metropolitan Perth (Whadjuk Boodjar). DYHSAC provides care to over 20,000 Aboriginal people in this area, servicing nearly two-thirds of the estimated 30,000 Aboriginal people living on Whadjuk Boodjar (*Australian Bureau of Statistics* 2016 Census).

The 1-week skin screening event took place in the October 2021 (spring) school holidays at the central DYHSAC site in East Perth. A large conference room adjoining the waiting room of DYHSAC was utilised for the study. Within this, three participant bays were created with moveable screens. A fourth bay with complete privacy was created for dermatology consultations, for those participants who transitioned to become patients requiring diagnosis and timely treatment. A children’s play zone was created in the centre of the room for supervised activities including play with bubbles and magnetic building toys, drawing and colouring, and singing and dancing to an educational culturally-appropriate YouTube Playlist created for this purpose (https://www.youtube.com/playlist?list=PLuiUTXzBzPh8MnaS-Xqh-RFaojFV5L3L3).

### Participants and recruitment

Promotion for the pilot project took place in collaboration with DYHSAC via clinic posters and social media. Participants were recruited from the DYHSAC waiting room. The parent or caregiver (hereafter ‘parent’) of the child attending DYHSAC was approached in the waiting room by a research team member and invited to participate. Where possible, Aboriginal members of the research team conducted this activity to improve cultural safety [29].

Aboriginal children 18 years and under were eligible for inclusion. The 2019 Modified Monash (MM) category was used to classify geographical remoteness, with urban-living defined as those participants residing in either MM 1 (metropolitan areas) or MM 2 (regional centres) [30].

Written informed consent was obtained following the provision of a plain language patient information and consent form (PICF) detailing the study. The consent process allowed parents to opt in or out of the various project components-questionnaire, height/weight, examination of exposed skin, examination of a specific concern on covered skin, clinical photographs of skin conditions, and skin swabs of suspected BSI. The participant was also required to provide verbal assent to the skin examination.

The skin screening week was staffed by a team of clinicians with dermatology and infectious disease expertise, Aboriginal Health Practitioners (AHP) and research support staff. All research team members attended Aboriginal cultural awareness and Good Clinical Practice training prior.

### Data collection

After enrolment, a research team member completed a questionnaire with the parent. Data including demographics, household structure and close contacts, access to household health hardware, history of skin infection, history of eczema, parent-reported Fitzpatrick skin phototype (FSP), sun exposure and sun-protective behaviours, skin care routine, use of traditional medicinal plants for skin care and skin disease, and current skin/hair/nail concerns was collected. To improve the validity of skin infection reports, clinical photos were shown to parents [31].

Following verbal assent, height, weight and body mass index were recorded and exposed (visible) skin, hair and nails examined. For participants with a skin concern on a covered site, examination was facilitated with privacy. Clinical photos of skin conditions and skin swabs for suspected BSI were taken.

Referral to the DYHSAC paediatric dermatology out-reach clinic was offered to those with an identified skin disorder; with same-day assessment provided for children with a skin infection or symptomatic disease. Education on *moorditj healthy skin* was provided to participants and their families using a purpose-built educational flipchart. Participants were remunerated for their time with a pool voucher and skin care products promoting *moorditj healthy skin*.

### Statistical analysis

All data was entered into REDCap and analysed using R version 4.1.2. Summary statistics of patient demographics and dermatological concerns were calculated. Logistic regression was used to investigate variations in the prevalence of BSI, dermatophyte infection, and atopic dermatitis (AD) in urban-living children by household structure, health hardware, frequency of bathing, use of bathing agent, frequency of swimming, past medical history and skin examination findings. Prevalence odds ratios and 95% confidence intervals investigating disease associations for AD, BSI and dermatophyte infection were calculated.

### Ethics

Ethics approval for this study was provided by the Western Australian Aboriginal Health Ethics Committee (WAAHEC) [HREC Ref No. 1059] and the University of Western Australia [File Reference–2021/ET000536].

## Results

### Feasibility measures

#### Promotion

The pilot project protocol limited the promotion of the screening week to activities associated with DYHSAC. Unfortunately, the major promotional event (The DYHSAC Family Fun Day) was cancelled due to COVID-19 lockdowns and poor weather. During the screening week the DYHSAC communications team utilised social media platforms to engage clients, and while data describing the avenue by which families heard about the screening week/were recruited was not collected, we suspect this online promotion was highly effective. Promotional posters produced by the research team were placed in the DYHSAC clinics.

#### Recruitment

From 4 to 8 October 2021, we recruited 84 children from the DYHSAC waiting room, 83 who identified as Aboriginal and 1 as Aboriginal and Torres Strait Islander. The recruited children were attending DYHSAC for varied reasons; some were there for their own appointment with their primary care physician or for ‘well child services’ (i.e. immunisations) with a nurse or AHP, others were accompanying a family member for their appointment, and some families had presented with their children having heard about the skin screening event through promotion or word-of-mouth.

A steady increase in participant numbers was observed over the course of the week with highest numbers recorded on the final day (*n* = 32, 38%). Although the enrolment rate was not formally recorded, almost all families approached in the waiting room subsequently presented for enrolment in the study room. As participant numbers increased over the course of the week, families tended to approach the study room to self-enrol rather than wait to be approached and recruited from the waiting room.

#### Data collection

All parents (84/84, 100%) consented to the major components of the project (questionnaire and examination) and all bar one child (83/84, 99%) provided verbal assent for examination. On average, the questionnaire with parents took up to ten minutes per child to complete. During this time, most children enjoyed play-based activities supervised by research support staff. For families with multiple children, paper-based questionnaires were preferred as they enabled research staff to record answers simultaneously for multiple children on separate documents.

On average, examination of exposed skin took less than five minutes per child to complete. Clinical photos were taken of skin conditions in 68% of consenting participants (55/81) and swabs from bacterial skin infections in 5% of consenting participants (4/79). Education was provided for families tailored to their child and the skin concerns highlighted during the questionnaire and/or examination, and complemented by the educational flipchart. Based on skin examination findings, nearly half of all participants (36/83, 43%) were referred to the paediatric dermatology outreach clinic; with 22/36 (61%) seen on the same day and 14/36 (39%) scheduled for later appointments.

#### Staffing

Staffing for the screening week varied from five to ten members each day; including at least one AHP and one dermatologist, in addition to other clinicians and research support staff. In total, seven clinical and nine non-clinical research staff were involved in the screening week.

#### Feedback and amendments

Following the pilot week, the screening team presented their observations, reflections and the results to the Elder and DYHSAC co-researchers, as well as the TKI Aboriginal Health Research Forum. Feedback was welcomed and from this, adjustments were made to the protocol to:

- Reflect ongoing Elder co-researcher cultural guidance and community consultation, ensuring cultural appropriateness and use of appropriate language and imagery.
- Expand the scope of promotion for subsequent screening weeks to minimise selection bias towards children with skin disease.
- Better inform the generalisability of results by including a question enquiring how families heard about the skin screening event.
- Enhance the validity of the results with the addition of a validated eczema symptom questionnaire to improve diagnostic precision (International Study of Asthma and Allergies in Childhood criteria–ISAAC) [32]; switching from parent-determined FSP to dermatologist-determined FSP enabling clarification of responses to more accurately classify FSP [33–36], and including validated measures of sun exposure and sun protection practices [37, 38].
- Further explore the preliminary hypotheses of disease associations with the addition of specific questions related to the reported predictive and protective factors.
- Optimise research document usability and statistical analysis for the research team.

The Koolungar Moorditj Healthy Skin project was awarded funding for expansion of the project to an additional urban site, and to support the co-design and development of skin health promotion and educational resources with Elders, AHPs and community advisory groups. An ethics amendment reflecting these changes was recently approved and this updated protocol will be followed for the planned multisite observational studies.

#### Dissemination of results

A one-page summary of the pilot project results was presented to the DYHSAC Board and staff (Additional file 5), and a thank-you letter with summarised results was shared with the participating families (Additional file 6). The findings have been co-presented at community forums, as well as local, national and international conferences. 

### Initial descriptions of skin health and skin disease frequency

#### Participant characteristics

The pilot project included 84 Aboriginal children (Table 1). The median age was 8 years (IQR 5–12) and 47/84 (56%) were female. Eighty of the 84 children (95%) were urban-living and contribute to this analysis.

**Table 1:** Participant demographics

	**Total (*n* = 84)**
Indigenous status	
- Aboriginal	83 (99%)
- Aboriginal and Torres Strait Islander	1 (1%)
Geographical location of usual residence	
- Metropolitan	80 (95%)
- Regional (inner/outer)	0
- Rural	0
- Remote	0
- Very remote	4 (5%)
Sex	
- Male	37 (44%)
- Female	47 (56%)
Age group	
- 0–9 years	49 (58%)
- 10–19 years	35 (42%)
Median age (IQR)	8 years (5, 12)

#### Questionnaire findings

Over half of all parents (45/80, 56%) reported a current concern with their child’s skin, hair and/or nails and one-third (26/80, 33%) described current itchy skin.

Aboriginal traditional medicines were reported as part of the child’s everyday skincare routine in 15% (12/80), and for treatment of skin problems in 21% (17/80). Only topical preparations were described, including plant extracts and animal (emu, goanna) oils.

FSP was parent-reported for 79 participants (Table 2). A history of past sunburn was present in 67% (53/79), with the highest rates in FSP II (4/5, 80%) and III (15/18, 83%). In FSP II and III, past sunburn was frequent with 6/53 (11%) reporting more than 10 sunburns. Most participants reported using sunscreen in the summer months (45/79, 57%), with 27% (21/79) never using sunscreen.

**Table 2:** Fitzpatrick skin phototype (parent-reported) and sun-protective behaviours in urban-living Aboriginal children

	**Total**^**a**^	**Fitzpatrick skin phototype (FSP)**^**b**^				
		**II**	**III**	**IV**	**V**	**VI**
***n* (%)**^**c**^	79	5 (6%)	18 (23%)	31 (39%)	22 (28%)	3 (4%)
**No history of past sunburn**	26 (33%)	1 (20%)	3 (17%)	11 (35%)	8 (36%)	3 (100%)
**History of past sunburn**	53 (67%)	4 (80%)	15 (83%)	20 (65%)	14 (64%)	0
< 10 times	46 (87%)	2 (50%)	11 (73%)	19 (95%)	14 (100%)	0
> 10 times	6 (11%)	2 (50%)	4 (27%)	0	0	0
Unknown frequency	1 (2%)	0	0	1 (5%)	0	0
**Frequency of sunscreen use**						
Daily to weekly	12 (15%)	2 (40%)	4 (22%)	2 (7%)	4 (18%)	0
Just in summer	45 (57%)	3 (60%)	12 (67%)	15 (48%)	14 (64%)	1 (33%)
Never	21 (27%)	0	2 (11%)	14 (45%)	4 (18%)	1 (33%)
Unknown frequency	1 (1%)	0	0	0	0	1 (33%

^a^*n* (%), percentages are column percentages. One child (aged 40 days old) had a missing value recorded for their FSP—they are excluded from this summary table. No participants had FSP Type I

^b^*n* (%), percentages are column percentages

^c^Row-wise percentages provided for this row

The lifetime prevalence of BSI, dermatophyte infection and scabies were 43% (34/80), 38% (30/80) and 14% (11/80), respectively (Table 3). A history of bone or joint infection was reported in 2/34 (6%) children with past BSI. No reports of sepsis or other serious post-infectious sequelae were found. The lifetime prevalence for eczema/dermatitis was 19% (15/80).

**Table 3:** Summary of past medical history in urban-living Aboriginal children

**“Has your child ever had …”**	**Total (*n* = 80)**
Skin infections	
- Bacterial skin infection (i.e. impetigo, skin sores, folliculitis, boils, cellulitis, abscess)	34 (43%)
- Fungal skin/hair/nail infection (i.e. tinea, ringworm)	30 (38%)
- Scabies	11 (14%)
Complications from skin infections	
- Bone or joint infection	2 (3%)
- Blood infection	0
- Acute rheumatic fever / Rheumatic heart disease	0
- Acute post-streptococcal glomerulonephritis	0
Atopy	
- Eczema/dermatitis	15 (19%)
- Hayfever	13 (16%)
- Asthma	9 (11%)
Iron deficiency	13 (16%)

#### Skin examination findings (Fig. 1a–f)

**Fig. 1:**
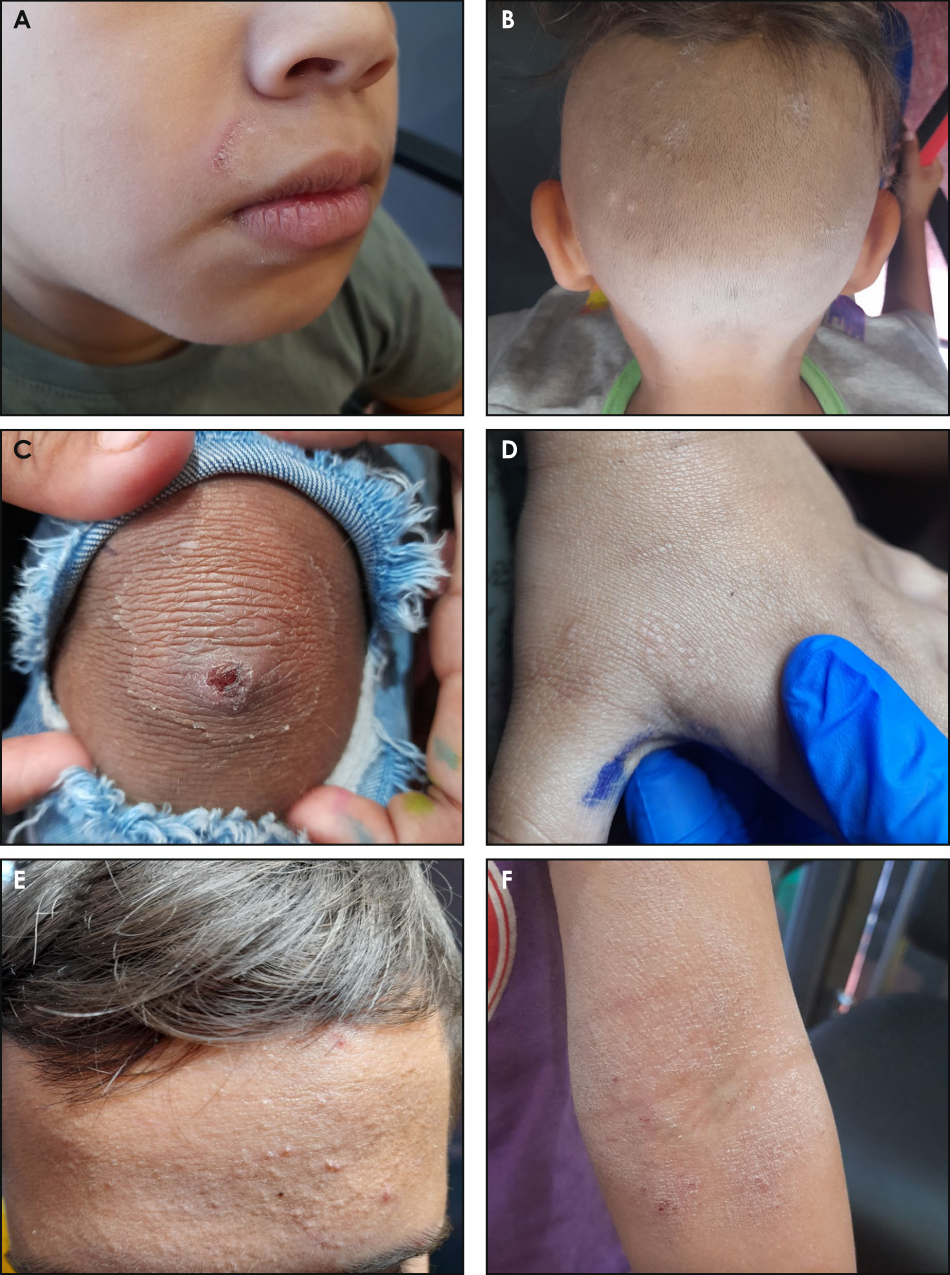
Skin examination findings. A Tinea faciei. B Tinea capitis. C Impetigo. D Scabies. E Acne. F Atopic dermatitis

The point prevalence was 23% (18/79) for pediculosis capitis, 19% (15/79) for dermatophyte infection, 5% (4/79) for BSI and 1% (1/79) for scabies (Table 4). Of eight children with tinea capitis, four also had tinea corporis, one was on appropriate oral treatment and *Trichophyton tonsurans* was cultured from all (6/6) of the adequately collected hair pluck specimens. Of the four participants with BSI (all were culture positive for *S. aureus*), two had a secondary infection of nipple atopic dermatitis, one had a knee furuncle (concurrent with headlice, tinea capitis and tinea corporis) and one had facial impetigo (concurrent with tinea corporis).

**Table 4:** Summary of skin examination findings in urban-living Aboriginal children

	**Total (*n* = 79**^**a**^**)**
Pediculosis capitis	18 (23%)
Acne	18 (23%)
Dermatophyte infections (all)	15 (19%)
- Tinea corporis	9 (11%)
- Tinea capitis	8 (10%)
Atopic dermatitis	12 (15%)
Seborrhoeic dermatitis	11 (14%)
Pityriasis alba	10 (13%)
Keratosis pilaris	8 (10%)
‘Other eczema/dermatitis’ (*incl*. irritant contact dermatitis, dyshidrotic eczema, juvenile plantar dermatosis, lip-lickers dermatitis)	6 (8%)
Acral naevi	5 (6%)

^a^*n* (%); (1 participant declined examination)

Among 10- to 19-year-olds, untreated acne was present in 55% (18/33) and seborrhoeic dermatitis in 18% (6/33). Acne phenotype was mostly comedonal and papulopustular, with prominent post-inflammatory hyperpigmentation. AD was diagnosed clinically in 15% (12/79) across all age groups. Pityriasis alba was seen in 12% (10/79), all of whom were FSP IV to VI. Keratosis pilaris was seen in 10% (8/79) and other types of eczema/dermatitis in 8% (6/79). Numerous other dermatological disorders were detected in one or two participants only, and the presence of naevi on exposed skin was recorded. There were no cases of childhood autoimmune disorders, including vitiligo, alopecia areata or cutaneous lupus. One child was subsequently diagnosed with hypopigmented mycosis fungoides. 

### Hypothesis-generating skin disease associations (Additional file 7)

#### Atopic dermatitis (AD)

A history of iron deficiency was associated with a fivefold increased risk (OR 5.2; 95% CI 1.3–21.2) of currently having AD.

#### Bacterial skin infections (BSI)

Participants with a history of low birth weight, iron deficiency and dermatophyte infection had 11-fold (OR 10.9; OR 1.3–94.1), fivefold (OR 5.5; 95% CI 1.3–22.9) and threefold (OR 3.4; 95% CI 1.3–8.9) risk of ‘ever’ having BSI, respectively. Children who reported ‘sometimes’ swimming in a chlorinated pool had an 83% reduction (OR 0.17; 95% CI 0.02–0.74) of ‘ever’ having BSI compared to those who reported ‘never’ swimming in a chlorinated pool.

#### Dermatophyte infections

Children who bed-share had sixfold (OR 5.9; 95% CI 1.5–22.8) chance of current dermatophyte infection and threefold (OR 2.9; 95% CI 1.1–7.4) chance of ‘ever’ having dermatophyte infection compared to participants who do not report bed-sharing. Children with current pediculosis capitis had threefold (OR 3.4; CI 1.1–10.1) greater odds of ‘ever’ having a dermatophyte infection. Children who reported ‘sometimes’ swimming in the ocean and those with a working washing machine at home had an 83% (OR 0.17; 95% CI 0.02–0.74) and 91% (OR 0.09; CI 0.01–0.75) reduction of ‘ever’ having dermatophyte infection, respectively.

## Discussion

This pilot study is the first assessment of skin health for urban-living Aboriginal children in Australia. We found it was feasible and informed subsequent larger studies underway. Aboriginal Elder co-design; involvement of Aboriginal researchers; and the research-service model were critical elements of success in the pilot. Historically, non-Aboriginal researchers have studied Aboriginal children and families. In this model, both Aboriginal and non-Aboriginal researchers walk together with Aboriginal children and families to discover answers to questions that are prioritised together.

Co-design is a “philosophical approach and evolving set of methodologies for involving people in the design of the services, strategies, environments, policies and processes that impact them’[39]. When practiced well, co-design in Indigenous health research can lead to outcomes that can be used to advocate for policy and service delivery changes, helping achieve equity [39, 40]. Co-design is recommended in research with Indigenous peoples in recognition of the history and wisdom of Aboriginal peoples, the longest, continuous culture in the world dating back more than 65,000 years [41]. Working together from a strengths-based approach to co-create Aboriginal-led systems and services supports self-determination and overcomes inequities that are a result of colonisation and racism [42]. The Koolungar Moorditj Healthy Skin project was developed through extensive consultation and cultural guidance from Noongar Elder co-researchers, and in partnership with DYHSAC; collaborating on all aspects of project development from needs assessment to content development, pilot testing and through to research dissemination [43].

The involvement of Aboriginal investigators in all roles of the research team was crucial to the success of the pilot project. In particular, the involvement of a trusted AHP embedded in DYHSAC was key to recruiting Aboriginal families, achieving a sample size of 84 participants and surpassing the estimated pilot size of 50. The value of a week-long screening event was evident in the observed increase in participant numbers over the course of the week peaking on the final day, which is consistent with cultural protocol and awareness. Integral to the study was establishing culturally appropriate dermatological care for urban-living Aboriginal children in a place where they feel most comfortable receiving healthcare. The ACCHO setting ensured cultural safety and facilitated the research-service model, whereby over a quarter of participants received opportunistic same-day management of their skin condition.

The results of the pilot project provide the first description of skin health and skin disease frequency in urban-living Aboriginal children recruited from the waiting room of an urban ACCHO. Recently, a systematic review has synthesised the available global literature on skin health in urban-living Indigenous children in high-income countries that share a history of colonisation, displacement and subsequent ongoing health inequities [19, 44]. Our results are comparable to the findings, and add further knowledge to the burden of these skin diseases, whilst scoping out a methodology for future studies.

This pilot project also acts as a hypothesis-generating study for associations of the more common childhood skin diseases. Using internal comparison to reduce bias, a number of possible disease associations were suggested.

### Infectious skin diseases

For BSI and scabies, we found a significantly lower prevalence in this urban-living cohort (5% and 1%, respectively) compared to the median prevalence reported for remote-living Aboriginal children (44.5% and 35%, respectively) [4, 5]. *S. aureus* was the only species cultured from BSI swab specimens in our pilot, while in the remote setting impetigo is mainly driven by *S. pyogenes*, with *S. aureus* playing a secondary role [45]. We found a history of iron deficiency was associated with a greater odds of ever having BSI. To our knowledge this association has not previously been described in Aboriginal children; however, both conditions occur more frequently in children where health inequities exist, including Indigenous, refugee and immigrant children [46, 47]. In the pilot cohort, low birthweight was associated with a greater odds of ever having BSI, consistent with an earlier study of WA Aboriginal children that demonstrated low birthweight to be a risk factor for skin infection hospitalisation [48].

In this pilot cohort, there appeared to be a high burden of pediculosis capitis (23%) and dermatophyte infection (19%), however background prevalence in urban Australian children is unknown. We identified a point prevalence of 11% for tinea capitis, which is the most infectious and most common paediatric dermatophyte infection globally, especially in low-income and resource-poor settings [49, 50]. This compares with up to 20% of the general population in developing countries and more than 30% of children in some urban areas of the USA [51]. *T. tonsurans* was the only species cultured from hair pluck specimens in our cohort. This anthropophilic species has historically been one of the most common species responsible for tinea capitis in Australia and is currently the most common cause of tinea capitis in the USA [52, 53].

Clustering of skin infections (dermatophyte infection, BSI and pediculosis capitis) was suggested in this pilot study. These skin infections are among the most common childhood skin disorders globally and co-infection is frequent, particularly in settings where poor housing conditions and health infrastructure exist, leading to ongoing transmission [31, 54]. A recent narrative review on systemic housing-level contributions to infectious disease transmission for Indigenous Australians found skin infections were associated with malfunctioning health hardware, including infrastructure required to wash clothes and bedding [55]. Consistent with this, the absence of a working washing machine was predictive of dermatophyte infection in our study. It has been shown that domestic laundering at 60 °C (as opposed to 30 °C) is required to completely eliminate dermatophytes; these temperatures are unlikely to be achieved with hand-washing of clothing, inability to afford heating of water for washing clothes, or a malfunctioning washing machine [56]. If this disease association is reproducible in the larger multisite observational studies, advocacy to address this modifiable factor in housing infrastructure programs may help reduce the burden of dermatophyte infection in urban-living Aboriginal children.

An interesting hypothesis-generating result in our study found that bed-sharing, defined as sharing a mattress with another person/people, was also predictive of dermatophyte infection. This can be explained by the contagious nature of dermatophyte infection, with spread known to occur from close contact with an infected person or contaminated bedding/clothing. Our pilot study failed to collect information on the reasons for bed-sharing, which is crucial for interpreting this result. It is known that sleeping arrangements are strongly influenced by cultural tradition, with bed-sharing reported to be one of the parental choices most influenced by cultural practice and beliefs [57, 58]. In many Aboriginal communities, purposeful bed-sharing is a cultural norm; however, bed-sharing may also be reactive and the result of environmental influences including socioeconomic factors, housing arrangements, heating and economic access to beds [57, 59, 60]. We hope the results of the larger multisite observational studies will provide the information needed to better translate this result, be it with advocacy for adequate housing infrastructure and/or empowering the community to consider health promotion messages that are appropriate and culturally strong.

Regarding protective factors for skin infections, the data suggest ‘sometimes’ (as compared with ‘never’) swimming in the ocean and ‘sometimes’ (as compared with ‘never’) swimming in a chlorinated pool may protect against dermatophyte infection and BSI, respectively. This latter result is supported by a systematic review investigating the health benefits of swimming pools in remote Aboriginal communities, in which the included prospective studies consistently demonstrated reduced skin sore prevalence associated with access to swimming pools [61].

### Non-infectious skin diseases

We found the lifetime prevalence of parent-reported ‘eczema ever’ (19%) in the pilot cohort to be less than that documented in all Australian children by age 4 years (28%), and less than that recently reported in a systematic review of all urban-living Indigenous children in high-income countries (median prevalence 25%) [15, 19]. Current AD was dermatologist-diagnosed on examination in 15% in our cohort (0–18 years); which, after taking into account the natural history of AD and its tendency to improve over time, is not dissimilar to the 20% population prevalence of clinician-diagnosed AD described in urban Australian children [14]. A history of iron deficiency was predictive for current AD in the pilot, consistent with evidence indicating adequate iron status in the perinatal and infantile period may protect against AD [62].

Untreated acne was detected in over half (55%) of all 10–19-year-olds in this pilot. An Australian population-based study has previously shown acne to be a common problem in school students, with prevalence ranging from 28% in 10–12-year-olds to 93% in 16–18-year-olds [63]. While little is known about the prevalence of acne in Aboriginal youth, a cross-sectional analysis of data from primary care reported a lower frequency of acne presentations by Aboriginal youth; suggesting either a lower incidence of acne or lower rates of Aboriginal youth seeking medical care for acne [64]. Further, among Australian studies profiling the case-mix of patients seen in various dermatology outpatient clinics, presentation for acne was uncommon in Aboriginal patients [21, 22, 65].

To our knowledge, this pilot data represents the first attempt to describe sun-protection behaviour and sunburn in urban-living Aboriginal children. Using the FSP classification, which is the most commonly used strategy to assess skin sensitivity to ultraviolet radiation despite inconsistencies being described in the skin of colour, we identified urban-living Aboriginal children with FSP II and III to be a subgroup with the most frequent sunburns where targeted sun protection messaging may be helpful [33, 34, 66–71]. This is further supported by research showing urban-living Indigenous adults with FSP II and III are at the highest risk of developing skin cancer, representing 19 of the 22 (86%) Indigenous patients diagnosed with skin cancer between 2003 and 2017 in Sydney, Australia [72].

### Strengths and limitations

The strengths of this study include its co-design with Noongar Elders, the research-service model embedded in an urban ACCHO, Aboriginal researchers involved in all study procedures, capacity building of AHPs, clinical diagnosis by a dermatologist and comprehensive data collection.

Limitations of this study include selection bias; most notably recruitment from a healthcare facility which may not represent all urban-living Aboriginal families therefore impacting on generalisability. Also, self-selection, in that parents of children with a dermatological concern may be more inclined to enrol their child skewing the results towards a greater level of disease.

Questionnaire responses carry the potential for recall bias and information bias may be present with the absence of validated questions for disease diagnosis (including eczema) and the absence of validated measures of sun exposure and sun protection practices. The FSP classification system has also been criticised for potential inconsistencies in the skin of colour, with some studies suggesting the questionnaire lacks reliability and functionality in this group [33, 34, 67, 68]. A better understanding of this topic in urban-living Aboriginal families is needed and may be a direction for future research.

The nature of this brief screening week taking place in spring means seasonal bias may be introduced for those identified skin conditions that show seasonal variation. Finally, the disease association results are limited by small sample sizes reflected in the wide confidence intervals. The findings of this pilot study must be considered in the context of these sources of potential bias.

## Conclusions

The Koolungar Moorditj Healthy Skin project is the first co-designed Australian study to describe skin health and disease in urban-living Aboriginal children. This pilot project was both feasible and acceptable, with high study participation and subsequent engagement in clinical care observed. Co-design and the strong involvement of Aboriginal people to lead and deliver the project was crucial. The successful pilot has enabled funding to be secured for a multi-site roll-out, where we hope to achieve an adequate sample size to better describe skin health and disease in this cohort, determine whether the preliminary hypotheses of association reported here are reproducible, and develop healthy skin messages for urban-living Aboriginal children in collaboration with Aboriginal Elders and community advisory groups.

### Supplementary Information

**Additional file 1.** CONSORT extension for Pilot and Feasibility Trials Checklist.

**Additional file 2.** STROBE checklist of items that should be included in reports of cohort studies.

**Additional file 3.** CONSIDER statement checklist of items to include when reporting research involving Indigenous Peoples.

**Additional file 4.** National Health and Medical Research Council (NHMRC) Indigenous Health Research Criteria.

**Additional file 5.** Summary to DYHSAC Board and Staff - KMHS October 2021 Screening Week.

**Additional file 6.** Thank You and Summary to Participants - KMHS October 2021 Screening Week.

**Additional file 7:** **Tables S5a, b, c.** Disease associations for AD, BSI and dermatophyte infection.

## Data Availability

The datasets generated and/or analysed during the current study are not publicly available due to Indigenous data sovereignty but are available from the corresponding author on reasonable request.

## Abbreviations

ACCHO: Aboriginal Community Controlled Health Organisation
AD: Atopic dermatitis
AHP: Aboriginal Health Practitioner
BSI: Bacterial skin infection
CONSIDER: CONSoliDated critERia
CONSORT: Consolidated Standards of Reporting Trials
DYHSAC: Derbarl Yerrigan Health Service Aboriginal Corporation
FSP: Fitzpatrick Skin Phototype
IQR: Interquartile range
ISAAC: International Study of Asthma and Allergies in Childhood
OR: Odds ratio
NHMRC: National Health and Medical Research Council
PICF: Patient Information and Consent Form
S. aureus: Staphylococcus aureus
S. pyogenes: Streptococcus pyogenes
STROBE: Strengthening the Reporting of Observational Studies in Epidemiology
T. tonsurans: Trichophyton tonsurans
TKI: Telethon Kids Institute
WAAHEC: Western Australian Aboriginal Health Ethics Committee
