# Supplementary material for: Describing skin health and disease in urban-living Aboriginal children: co-design, development and feasibility testing of the Koolungar Moorditj Healthy Skin pilot project
Source: Pilot Feasibility Stud. 2024 Jan 11;10:6. doi: 10.1186/s40814-023-01428-6 (PMC10782716; doi:10.1186/s40814-023-01428-6)
Supplement: Supplementary file 4 — Additional file 4. National Health and Medical Research Council (NHMRC) Indigenous Health Research Criteria. [file 40814_2023_1428_MOESM4_ESM.pdf]

Australian Government
National Health and Medical Research Council

NHMRC

SAPPHIRE

Indigenous Research Excellence Criteria

Application Details	
**Grant Opportunity:**	2021 Postgraduate Scholarships
**Application ID:**	2014208
**Application Title:**	Koolungar Urban Moorditj Healthy Skin: Determining the burden of skin disease in urban Aboriginal children and adolescents.
**Chief Investigator A:**	Dr Bernadette Ricciardo
**Administering Institution:**	University of Western Australia
**Grant Duration:**	3 years

Aboriginal and/or Torres Strait Islander Research Excellence Criteria

Community Engagement

Community Engagement

The Koolungar Urban Moorditj Healthy Skin project has been co-designed from the outset with Noongar Elders to determine the interest, scope and importance of skin disease for urban Aboriginal children. Community engagement began in 2019 with several discussions with the Ngulluk Koolunga, Ngulluk Koort (Our Children, Our Heart) Elder/Co-researcher group. Over the course of the last year the project has been co-designed with Uncle Noel Nannup and Aunty Dale Tilbrook, who will have continued involvement as Elder/Co-researchers during the implementation phase, with support from the Aboriginal Health and Wellbeing team at Telethon Kids Institute (TKI). A Seed Funding Grant from the Wesfarmers Centre of Vaccines & Infectious Diseases (WCVID) has created capacity for this partnership, facilitating conversations to shape the project.

Integral to the study is establishing culturally appropriate dermatological care for urban Aboriginal youth in a place where they feel most comfortable. This project is a collaboration with Derbarl Yerrigan Health Service (DYHS), the largest Aboriginal community-controlled health service in WA. The CEO of DYHS and the Research and Development Sub-Committee view our project as “vital in determining the burden of skin infections and skin disease in Aboriginal children and young people with the outcome of translating to healthy skin.” We have recently obtained a WCVID Capacity Building Grant to fund a DYHS Aboriginal Health Practitioner in our clinic, pilot project and co-design team.

This project has been presented to the TKI Aboriginal Health Research Forum, with their recommendations incorporated into the design. Following extensive consultation, the project has received ethics approval from the WA Aboriginal Health Ethics Committee. The knowledge and wisdom of Noongar Elders, researchers, and community members has been integral in project development and will continue to guide delivery and dissemination of the research findings.

Benefit

Benefit

BENEFITS TO THE PARTICIPANT, THEIR FAMILY, CLOSE CONTACTS & COMMUNITY -
Using a research-service model, participants will benefit directly from prompt management of their skin disease. In the case of contagious skin infection, the early management of the participant will prevent spread to their families, close contacts and community. Families and close contacts will also benefit from ‘healthy skin’ education provided as part of this project.

The involvement of a DYHS-employed Aboriginal Health Practitioner in our research team and clinic will provide an excellent opportunity for on-the-job dermatology training, with ongoing benefit to the community DYHS serves well beyond the life of the project.

The results of this project will define the burden of skin disease in the youth that DYHS services. This will inform DYHS about the contribution skin problems make to their overall activity, enabling the design of clinical referral pathways and services to better target this.

BENEFITS TO THE BROADER URBAN-LIVING AUSTRALIAN ABORIGINAL COMMUNITY -
The results of this project will inform the urban aspects of the National Healthy Skin Guideline 2nd Edition, leading to improved primary care management of skin infection in the urban setting to prevent hospitalisation and reduce long-term morbidity. The first edition of the National Healthy Skin Guideline was launched in 2018 by Minister Ken Wyatt (Minister for Indigenous Affairs) and is widely used across Australia for skin health assessments and management. It will be important to have more robust information included for urban living families in the second edition.

The results of this project will facilitate co-design of clinical trials to identify sustainable, community-wide strategies for prevention and treatment of skin disease. We expect the project findings will highlight the importance of healthy housing in preventing skin infection, leading to changes in housing policy that will directly benefit all urban Aboriginal people.

Sustainability and Transferability

Sustainability and Transferability

Our co-design framework demonstrates best practice for research with Aboriginal youth and their families. It recognizes equal value of Aboriginal and Western knowledge systems, nurturing two-way learning and collaboration. While the Noongar Elder/Co-researchers provide leadership to ensure the project is culturally safe and significant to the health of Aboriginal people, the Wadjella members ensure a high standard of research practice is maintained. This co-design framework is sustainable and transferable to all research in Aboriginal health.

The research-service model initiated will deliver on much needed service-provision while filling a knowledge gap. The involvement and teaching of Aboriginal health staff is crucial for service sustainability. I will provide teaching for Aboriginal medical students and doctors interested in pursuing a dermatology career, with our clinic creating an ideal setting for clinical exposure, research opportunities and mentorship. Such partnerships will build the Aboriginal specialist dermatology workforce to ensure sustainability of this clinic, with potential for this model to be transferred to other Aboriginal medical services.

Through co-design and considered research practices, I am committed to minimising cost to the Aboriginal participant, their family and community. Specifically, I will act to minimise the research burden on Aboriginal people, maintain intellectual property rights and ensure all engagements are culturally appropriate, inclusive and respectful.

Skin infections in urban Aboriginal children have a high burden on the health system, both in primary care and hospitals where urban Aboriginal children are 10 times more likely to be admitted for skin infection than their non-Aboriginal peers. This project will improve knowledge in urban Aboriginal families on how to maintain healthy skin, prevent and recognize skin infection; benefiting the individual, their family and community, with reduced cost to the health system.

Build Capacity

Build Capacity

This project has been developed and guided by the Elder/Co-researchers as leaders within the research team, acknowledged by the naming of the Elder/Co-researchers as investigators on the project. We will continue to create opportunities for Aboriginal people to actively participate in the project thereby building research capacity, and we will encourage input and feedback from participants and community members.

The research team holds reciprocity as a core value throughout the development and implementation of the project. We have built a collaborative and trusting research partnership with the DYHS staff and community. Employment of a DYHS Aboriginal Health Practitioner in our clinic and research team will enhance community engagement, ensuring the processes and outcomes of our project are aligned with the vision and priorities of DYHS and the families it serves. The involvement of a DYHS Aboriginal Health Practitioner will also provide an excellent opportunity for on-the-job dermatology teaching, which will be of ongoing benefit to the community DYHS serves. This is evidence of capacity building to align with the value of reciprocity. In addition, by teaching staff already embedded in DYHS rather than employing external staff, we are creating a sustainable model to ensure the dermatology knowledge taught is translated into clinical practice beyond the project.

This research project has the capability to provide mentoring for Aboriginal medical students and doctors interested in pursuing a career in Dermatology. Clinical observerships in the DYHS paediatric dermatology out-reach clinic are available as well as the opportunity to become involved in research, with the longer-term goal of building the Aboriginal specialist dermatology and research workforce.

This project provides an opportunity to improve knowledge of the importance of skin health amongst Aboriginal communities, as well as strengthen the community's capacity to manage skin disease in their community.
