# Supplementary material for: Describing skin health and disease in urban-living Aboriginal children: co-design, development and feasibility testing of the Koolungar Moorditj Healthy Skin pilot project
Source: Pilot Feasibility Stud. 2024 Jan 11;10:6. doi: 10.1186/s40814-023-01428-6 (PMC10782716; doi:10.1186/s40814-023-01428-6)
Supplement: Supplementary file 5 — Additional file 5. Summary to DYHSAC Board and Staff - KMHS October 2021 Screening Week. [file 40814_2023_1428_MOESM5_ESM.pdf]

To the Derbarl Yerrigan Health Service staff ….

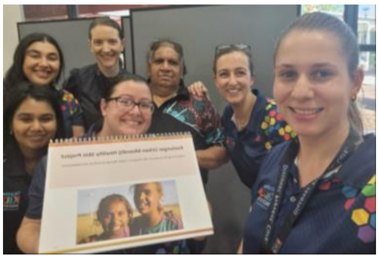

From all of us in the **Koolungar Moorditj Healthy Skin** research team, we would like to say a big **THANK YOU** for welcoming us into your space and promoting our pilot skin screening event in October 2021! We also acknowledge and thank the Noongar Elders for their continued cultural guidance and wisdom throughout this project.

Thanks to you, we now know a bit more about skin health in urban Aboriginal and Torres Strait Islander children. We have also learnt a few great tips for improving future healthy skin screening weeks!

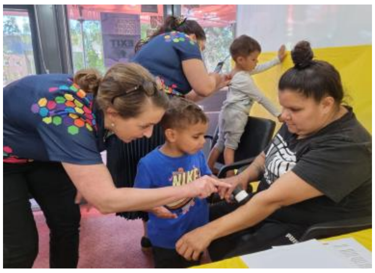

Here are some of the results of the screening week…
84 children (0 to 18 years) participated in the screening week.

- Just over half of participating children had a current concern with their skin, hair or nails.
- Around one third of participating children described current itchy skin.

From the questionnaire, we learnt that of the 84 participating children –

- 64% had previously been sunburnt.
- 43% had previously had impetigo (skin sores), with 6% having a serious complication from their bacterial skin infection requiring hospitalisation.
- 37% had previously had tinea (ring-worm).
- 18% had previously had atopic dermatitis (eczema).
- 13% children had previously had scabies.

We also learnt **bush medicines are commonly used for the skin**, with 1 in every 5 participating children using bush medicine as part of their everyday skincare, and over one quarter of children using bush medicine when they have a skin problem.

The most common **infectious skin conditions** on skin examination were –

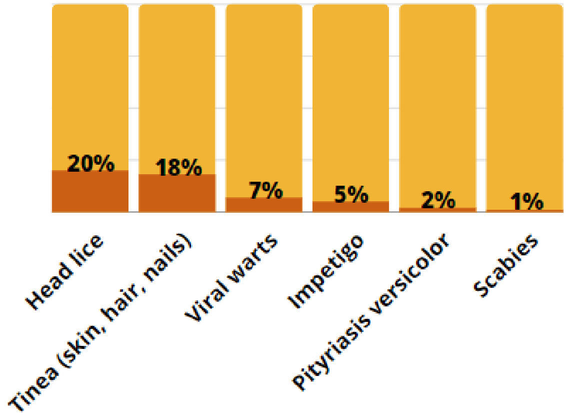

The most common **non-infectious skin conditions** on skin examination were –

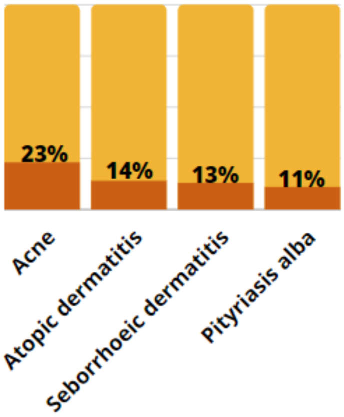

During the pilot skin screening week, our dermatologist, Dr Bernadette Ricciardo, provided treatment for 22 participating children with skin conditions. Another 14 children were referred to see Dr Bernie later in the dermatology clinic at Derbarl.

Thank you for helping us to learn more about skin health in urban Aboriginal and Torres Strait Islander children. We look forward to the next Koolungar Moorditj Healthy Skin screening week in 2022! And remember, **if your patient has a skin, hair, or nail concern – our dermatologist can see them at Derbarl, East Perth**.

Dr Bernadette Ricciardo and the Koolungar Moorditj Healthy Skin Team –
KoolungarMoorditjHealthySkin@telethonkids.org.au

Please note that in this document Aboriginal refers to both Aboriginal and/or Torres Strait Islander peoples. We recognise and acknowledge the strong diversity between Aboriginal and Torres Strait Islander cultures, and we do not intend to diminish any identity.
