# Supplementary material for: Describing skin health and disease in urban-living Aboriginal children: co-design, development and feasibility testing of the Koolungar Moorditj Healthy Skin pilot project
Source: Pilot Feasibility Stud. 2024 Jan 11;10:6. doi: 10.1186/s40814-023-01428-6 (PMC10782716; doi:10.1186/s40814-023-01428-6)
Supplement: Supplementary file 6 — Additional file 6. Thank You and Summary to Participants - KMHS October 2021 Screening Week. [file 40814_2023_1428_MOESM6_ESM.pdf]

To the children and families

From all of us in the **Koolungar Moorditj Healthy Skin** research team, we would like to say a big **THANK YOU** for taking part in the skin health screening week in October 2021! We also acknowledge and thank the Noongar Elders for their continued cultural guidance and wisdom throughout this project.

Background

We have lots of information on skin health and skin sores for children living in remote areas of WA, but we don't know much about skin health for Aboriginal koolungar (children) living in WA cities and towns.

This is important because untreated skin sores can be serious and can make you really sick. They can also spread between family and friends and cause itchiness, pain, shame, sleeplessness and poor concentration at school.

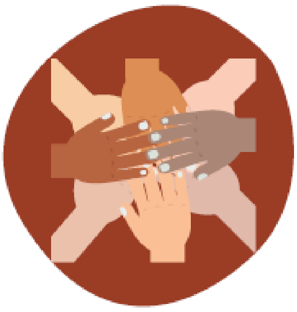

Your participation in the screening week has helped us learn more about skin health in Aboriginal children on Whadjuk Boodja and understand how we can improve our screening weeks – thank you!

Screening week

84 moorditj koolungar took part in the screening week at Derbarl Yerrigan.

Nearly half of the koolungar told us they had previously had skin sores, and some of these koolungar had needed to go to hospital because of serious sickness from their skin sores. However, during the skin checks, skin sores were only found in small number of koolungar.

We also looked at other itchy skin conditions that lead to scratching, making skin infection more likely. The most common of these conditions seen during the skin checks were headlice, ringworm (tinea), and eczema (atopic dermatitis).

We saw lots of really moorditj skin, with nearly half of all children reporting no problem with their skin, hair or nails. 1 in 5 children were using bush medicine as part of their everyday skincare and 1 in 4 used bush medicine when they had a skin concern.

During the week, our dermatologist provided same-day treatment for 22 children and another 14 were referred to the dermatology clinic at Derbarl to be seen later.

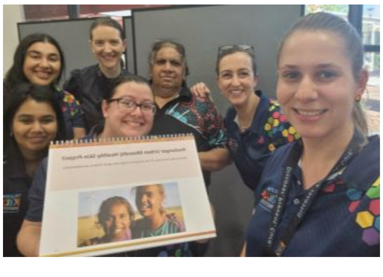

If you at all are worried about your child's skin, ask your GP for a referral to see our dermatologist at Derbarl Yerrigan in East Perth.

We look forward to the next Koolungar Moorditj Healthy Skin screening week in 2022!

Dr Bernadette Ricciardo and the Koolungar Moorditj Healthy Skin Team

KoolungarMoorditjHealthySkin@telethonkids.org.au

Please note that in this document Aboriginal refers to both Aboriginal and/or Torres Strait Islander peoples. We recognise and acknowledge the strong diversity between Aboriginal and Torres Strait Islander cultures, and we do not intend to diminish any identity.

Summary

From all of us in the **Koolungar Moorditj Healthy Skin** research team, we would like to say a big **THANK YOU** for taking part in the skin health screening week in October 2021! We also acknowledge and thank the Noongar Elders for their continued cultural guidance and wisdom throughout this project.

Here are some of the results of the screening week at Derbarl Yerrigan where **84 moorditj koolungar** (children) participated.

Of the 84 koolungar:

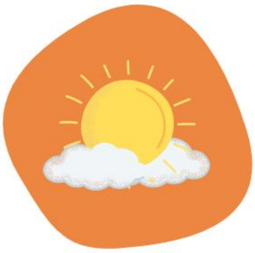

- 1 in 5 were using bush medicine as part of their everyday skincare and 1 in 4 used bush medicine when they had a skin problem.
- Over half (54%) use sunscreen in the summer months, while nearly one third (31%) never use sunscreen. 64% had previously been sunburnt.
- 43% had previously had skin sores, 6% of those had been to the hospital in the past because of a sickness from skin sores (infections). No child had rheumatic fever or rheumatic heart disease.
- 37% had previously had ring worm (tinea), affecting the skin, hair, or nails.
- 18% had previously had eczema, similar to rates seen in Australian non-Aboriginal children
- 13% had previously had scabies.

These are some of the conditions we found during the skin checks…

Source of images: dermnetnz.org/image-library

<u>Infectious</u>

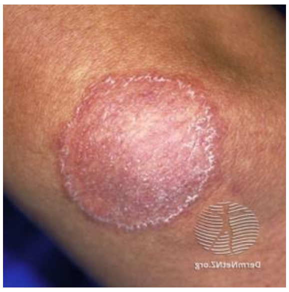

18% ringworm (tinea)

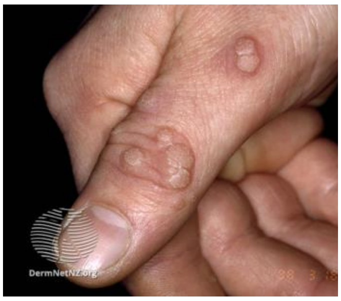

7% viral warts

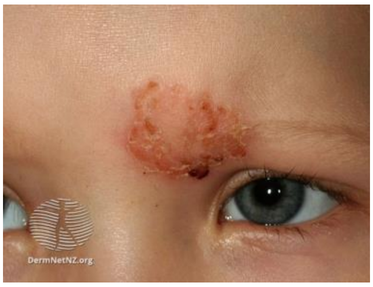

5% skin sores (impetigo)

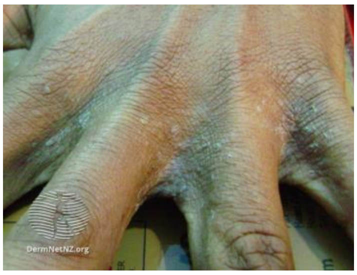

1% scabies

<u>Non-infectious</u>

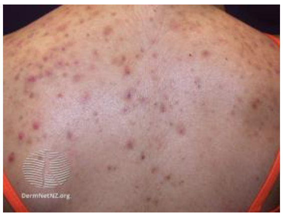

23% acne

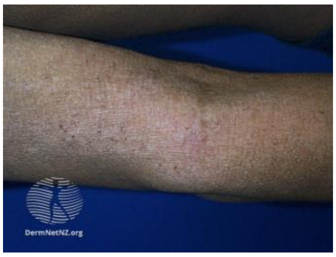

14% eczema (atopic dermatitis)

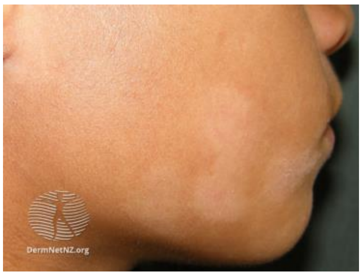

11% pityriasis alba

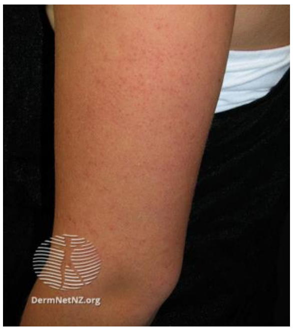

10% keratosis pilaris

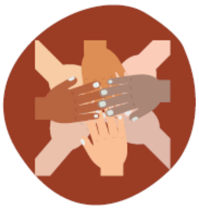

Thank you for helping us all learn more about skin health in Aboriginal children on Whadjuk Boodja.

Dr Bernadette Ricciardo and the Koolungar Moorditj Healthy Skin Team
KoolungarMoorditjHealthySkin@telethonkids.org.au

Please note that in this document Aboriginal refers to both Aboriginal and/or Torres Strait Islander peoples. We recognise and acknowledge the strong diversity between Aboriginal and Torres Strait Islander cultures, and we do not intend to diminish any identity.
