# Supplementary material for: Describing skin health and disease in urban-living Aboriginal children: co-design, development and feasibility testing of the Koolungar Moorditj Healthy Skin pilot project
Source: Pilot Feasibility Stud. 2024 Jan 11;10:6. doi: 10.1186/s40814-023-01428-6 (PMC10782716; doi:10.1186/s40814-023-01428-6)
Supplement: Supplementary file 7 — Additional file 7: Tables S5a, b, c. Disease associations for AD, BSI and dermatophyte infection. [file 40814_2023_1428_MOESM7_ESM.docx]

**Table 5a – Atopic dermatitis: disease associations**

| **Response category** | | **Ever eczema / dermatitis** (n = 15) | | | | **Current AD** (n = 12) | | | | | |
| --- | --- | --- | --- | --- | --- | --- | --- | --- | --- | --- | --- |
|  |  | n (%) | OR (95% CI) | | | n (%) | | | | OR (95% CI) | |
| ***Household structure*** | |  |  | |  | | |  | | |  |
| Household size >3 | | 10 (67%) | 0.36 (0.1, 1.3) | | | 10 (83%) | | | | 1.2 (0.23, 6.17) | |
| Stable household numbers | | 14 (93%) | 1.58 (0.18, 14.27) | | | 11 (92%) | | | | 1.08 (0.12, 9.88) | |
| Pets (cats and/or dogs) | | 7 (47%) | 0.6 (0.19, 1.88) | | | 6 (50%) | | | | 0.76 (0.22, 2.61) | |
| Bedsharing | | 3 (20%) | 0.2 (0.05, 0.77) | | | 5 (42%) | | | | 0.74 (0.21, 2.55) | |
| ***Household health hardware*** | |  |  | |  | | |  | | |  |
| Working washing machine at home | | 14 (93%) | 1.02 (0.11, 9.84) | | | 11 (92%) | | | | 1.08 (0.12, 9.88) | |
| Plumbing or bathroom maintenance issues at home | | 0 (0%) | - | | | 0 (0%) | | | | - | |
| ***Frequency of bathing*** | |  |  | |  | | |  | | |  |
| Frequent^#^ | | 15 (100%) | - | | 12 (100%) | | | - | | |  |
| ***Use of bathing agent*** | |  |  | |  | | |  | | |  |
| Any bathing agent^ | | 14 (93%) | 3.64 (9.14, 96.1) | | 11 (92%) | | | 5.36 (0.20, 142.7) | | |  |
| ***Swimming in a chlorinated pool*** | |  |  | |  | | |  | | |  |
| Sometimes^*^ | | 11 (73%) | 0.48 (0.12, 2.08) | | 9 (75%) | | | 0.49 (0.11, 2.58) | | |  |
| ***Swimming in the ocean*** | |  |  | |  | | |  | | |  |
| Sometimes^*^ | | 13 (87%) | 3.54 (0.83, 25.11) | | 11 (92%) | | | 5.94 (0.99, 116.03) | | |  |
| ***Perinatal history*** | |  |  | |  | | |  | | |  |
| Low birth weight | | 2 (13%) | 1.73 (0.3, 9.85) | | 2 (17%) | | | 2.15 (0.37, 12.33) | | |  |
| Maternal smoking in pregnancy | | 2 (13%) | 0.44 (0.09, 2.24) | | 4 (33%) | | | 1.55 (0.4, 5.96) | | |  |
| Maternal multiparity | | 4 (27%) | 0.73 (0.2, 2.65) | | 4 (33%) | | | 0.98 (0.26, 3.68) | | |  |
| Very remote location of birth | | 0 (0%) | - | | 0 (0%) | | | - | | |  |
| ***Past medical history*** | |  |  | |  | | |  | | |  |
| Bacterial skin infection | | 6 (40%) | 1.21 (0.36, 4.07) | | | 6 (50%) | | | | 1.69 (0.47, 6.11) | |
| Dermatophyte infection | | 9 (60%) | 3.16 (0.98, 10.16) | | | 7 (58%) | | | | 2.62 (0.75, 9.18) | |
| Scabies | | 3 (20%) | 1.56 (0.36, 6.79) | | | 3 (25%) | | | | 2.42 (0.54, 10.84) | |
| Iron deficiency | | 5 (33%) | 3.14 (0.83, 11.97) | | | **5 (42%)** | | | | **5.21 (1.28, 21.16)** | |
| Vitamin D deficiency | | 0 (0%) | - | | | 1 (8.3%) | | | | 3.06 (0.25, 37.29) | |
| Asthma | | 2 (13%) | 1.24 (0.23, 6.72) | | | 2 (17%) | | | | 1.84 (0.33, 10.3) | |
| Hayfever | | 5 (33%) | 3.64 (0.96, 13.81) | | | 2 (17%) | | | | 1.08 (0.21, 5.69) | |
| Food allergy | | 0 (0%) | - | | | 0 (0%) | | | |  | |
| ***Skin examination findings*** |  | | |  | | |  | |  | |  |
| Pediculosis capitis | | 5 (33%) | 2.45 (0.69, 8.73) | | | 3 (25%) | | | | 1.16 (0.28, 4.82) | |
| Bacterial skin infection | | 2 (13%) | 4.38 (0.56, 34.07) | | | 2 (17%) | | | | 6.5 (0.82, 51.51) | |
| Dermatophyte infection | | 1 (6.7%) | 0.23 (0.03, 1.9) | | | 0 (0%) | | | | - | |
| Scabies | | 1 (6.7%) | - | | | 0 (0%) | | | | - | |
|  | |  |  | | |  | | | |  | |

**Key**: Frequent^#^ - defined as twice daily, daily or second daily; as compared with weekly

Any bathing agent^ - including soap-free wash, soap or shower gel; as compared with water alone

Sometimes^*^ - defined as weekly, monthly or just in the summer; as compared with never

**Table 5b – Bacterial skin infection: disease assocations**

| **Response category** | **Ever BSI** (n = 33) | | **Current BSI** (n = 4) | |
| --- | --- | --- | --- | --- |
|  | n (%) | OR (95% CI) | n (%) | OR (95% CI) |
| ***Household structure*** |  |  |  |  |
| Household size >3 | 25 (76%) | 0.61 (0.2, 1.89) | 3 (75%) | 0.69 (0.07, 7.12) |
| Stable household numbers | 29 (88%) | 0.54 (0.11, 2.62) | 4 (100%) | - |
| Pets (cats and/or dogs) | 15 (45%) | 0.4 (0.16, 1.03) | 1 (25%) | 0.25 (0.02, 2.5) |
| Bedsharing | 20 (61%) | 2.14 (0.85, 5.39) | 2 (50%) | 1.08 (0.14, 8.1) |
| ***Household health hardware*** |  |  |  |  |
| Working washing machine at home | 30 (91%) | 1.03 (0.21, 4.93) | 3 (75%) | 0.26 (0.02, 2.91) |
| Plumbing or bathroom maintenance issues at home | 2 (6.1%) | 0.63 (0.11, 3.66) | 0 (0%) | - |
| ***Frequency of bathing*** |  |  |  |  |
| Frequent^#^ | 32 (97%) | 0.24 (0.01, 1.49) | 4 (100%) | - |
| ***Use of bathing agent*** |  |  |  |  |
| Any bathing agent^^^ | 33 (100%) | - | 3 (75%) | 0.22 (0.76, 674.2) |
| ***Swimming in a chlorinated pool*** |  |  |  |  |
| Sometimes^*^ | 31 (94.1%) | **0.17 (0.02, 0.74)** | 2 (50%) | 5.15 (0.55, 49.40) |
| ***Swimming in the ocean*** |  |  |  |  |
| Sometimes^*^ | 27 (82%) | 0.54 (0.16, 1.77) | 3 (75%) | 0.58 (0.03, 5.33) |
| ***Perinatal history*** |  |  |  |  |
| Low birth weight | 7 (21%) | **10.92 (1.27, 94.18)** | 0 (0%) | - |
| Maternal smoking in pregnancy | 11 (33%) | 1.52 (0.55, 4.23) | 1 (25%) | 1.28 (0.11, 14.86) |
| Maternal multiparity | 12 (36%) | 1.16 (0.44, 3.03) | 1 (25%) | 0.85 (0.07, 9.84) |
| Very remote location of birth | 0 (0%) | - | 0 (0%) | - |
| ***Past medical history*** |  |  |  |  |
| Dermatophyte infection | 18 (55%) | **3.38 (1.28, 8.93)** | 4 (100%) | - |
| Scabies | 4 (12%) | 0.73 (0.2, 2.76) | 0 (0%) | - |
| Eczema | 6 (18%) | 1.21 (0.36, 4.07) | 2 (50%) | - |
| Iron deficiency | 9 (27%) | **5.53 (1.33, 22.89)** | 1 (25%) | 1.47 (0.14, 15.41) |
| Vitamin D deficiency | 1 (3.0%) | 0.83 (0.07, 9.63) | 0 (0%) | - |
| ***Skin examination findings*** |  |  |  |  |
| Pediculosis capitis | 11 (33%) | 3.08 (1, 9.51) | 1 (25%) | 1.14 (0.11, 11.65) |
| Dermatophyte infection | 10 (30%) | 3.3 (1, 10.88) | 2 (50%) | 4.77 (0.61, 37.01) |
| Scabies | 0 (0%) | - | 0 (0%) | - |
| Atopic dermatitis | 6 (18%) | 1.69 (0.47, 6.11) | 2 (50%) | 6.5 (0.82, 51.51) |

**Key**: Frequent^#^ - defined as twice daily, daily or second daily; as compared with weekly

Any bathing agent^ - including soap-free wash, soap or shower gel; as compared with water alone

Sometimes^*^ - defined as weekly, monthly or just in the summer; as compared with never

**Table 5c – Dermatophyte infection: disease associations**

| **Response category** | | **Ever dermatophyte** (n = 30) | | | | | **Current dermatophyte** (n = 15) | |
| --- | --- | --- | --- | --- | --- | --- | --- | --- |
|  | | n (%) | | | OR (95% CI) | | n (%) | OR (95% CI) |
| ***Household structure*** | | |  |  | |  | |  |
| Household size >3 | | 23 (77%) | | | 0.56 (0.17, 1.8) | | 11 (73%) | 0.57 (0.15, 2.13) |
| Stable household numbers | | 26 (87%) | | | 0.43 (0.09, 2.09) | | 13 (87%) | 0.55 (0.1, 3.16) |
| Pets (cats and/or dogs) | | 14 (47%) | | | 0.57 (0.23, 1.44) | | 7 (47%) | 0.64 (0.21, 1.98) |
| Bedsharing | | 19 (63%) | | | **2.88 (1.12, 7.41)** | | 12 (80%) | **5.85 (1.5, 22.78)** |
| ***Household health hardware*** | | |  |  | |  | |  |
| Working washing machine at home | 24 (80%) | | | | **0.09 (0.01, 0.75)** | | 13 (87%) | 0.55 (0.1, 3.16) |
| Plumbing or bathroom maintenance issues at home | 3 (10%) | | | | 1.67 (0.31, 8.85) | | 1 (6.7%) | 0.84 (0.09, 7.8) |
| ***Frequency of bathing*** | | |  |  | |  | |  |
| Frequent^#^ | | | 30 (100%) | - | | 15 (100%) | | - |
| ***Use of bathing agent*** | | |  |  | |  | |  |
| Any bathing agent^ | | | 29 (97%) | 1.38 (0.05, 35.8) | | 15 (100%) | | - |
| ***Swimming in a chlorinated pool*** | | |  |  | |  | |  |
| Sometimes^*^ | | | 23 (76.3%) | 2.55 (0.67, 11.23) | | 14 (93%) | | 0.24 (0.01, 1.39) |
| ***Swimming in the ocean*** | | |  |  | |  | |  |
| Sometimes^*^ | | | 27 (90%) | 0.17 (0.03, 0.63) | | 11 (73%) | | 1.36 (0.32, 5.18) |
| ***Perinatal history*** | | |  |  | |  | |  |
| Low birth weight | | | 3 (10%) | 0.92 (0.2, 4.2) | | 1 (6.7%) | | 0.59 (0.07, 5.25) |
| Maternal smoking in pregnancy | | | 11 (37%) | 2.59 (0.9, 7.42) | | 6 (40%) | | 2.25 (0.67, 7.54) |
| Maternal multiparity | | | 8 (27%) | 0.5 (0.18, 1.35) | | 2 (13%) | | 0.23 (0.05, 1.12) |
| Very remote location of birth | | | 0 (0%) | - | | 0 (0%) | | - |
| ***Past medical history*** | | |  |  | |  | |  |
| Bacterial skin infection | | 18 (60%) | | | **3.38 (1.28, 8.93)** | | 10 (67%) | 3.3 (1, 10.88) |
| Scabies | | 7 (23%) | | | - | | 1 (6.7%) | 0.42 (0.05, 3.54) |
| Eczema | | 9 (30%) | | | 3.5 (0.92, 13.24) | | 1 (6.7%) | 0.23 (0.03, 1.9) |
| Iron deficiency | | 5 (17%) | | | 1.28 (0.37, 4.49) | | 1 (6.7%) | 0.38 (0.04, 3.3) |
| Vitamin D deficiency | | 0 (0%) | | | - | | 0 (0%) | - |
| ***Skin examination findings*** | |  | | |  | |  |  |
| Pediculosis capitis | | 11 (37%) | | | **3.39 (1.14, 10.11)** | | 6 (40%) | 2.89 (0.86, 9.68) |
| Bacterial skin infection | | 4 (13%) | | | - | | 2 (13%) | 4.77 (0.61, 37.01) |
| Scabies | | 1 (3.3%) | | | - | | 0 (0%) | - |
| Atopic dermatitis | | 7 (23%) | | | 2.62 (0.75, 9.18) | | 0 (0%) | - |

**Key**: Frequent^#^ - defined as twice daily, daily or second daily; as compared with weekly

Any bathing agent^ - including soap-free wash, soap or shower gel; as compared with water alone

Sometimes^*^ - defined as weekly, monthly or just in the summer; as compared with never
